# Predicting Factors of Depression, Antidepressant Use and Positive Response to Antidepressants in Perinatal and Postpartum Women

**DOI:** 10.2174/1745017901713010049

**Published:** 2017-06-30

**Authors:** Huyen Vu, Fadia T. Shaya

**Affiliations:** 1 University of Maryland School of Pharmacy, Department of Pharmacy Practice and Science, Baltimore, MD, USA; 2 University of Maryland School of Pharmacy, Department of Pharmaceutical Health Services Research Baltimore, MD, USA

**Keywords:** Depression, Antidepressant, Perinatal women, Postpartum women, Illicit drug

## Abstract

**Background:**

In the United States, there is a disparity in knowledge of nationwide depression prevalence, the antidepressant use and the antidepressant responses during perinatal/postpartum periods.

**Objective:**

This study investigated the predicting factors of depression, antidepressant use and positive antidepressant response during the perinatal/postpartum periods.

**Method:**

The 2007-2012 National Health and Nutrition Examination Surveys (NHANES) were combined to identify adult pregnant women, those within the 18-month postpartum period (n=492) and their depression statuses *via* demographics, health care accessibility, antidepressant use and illicit drug use information. The characteristics of different study groups were compared (depression versus no-depression groups, antidepressant users versus non-antidepressant users, and antidepressant responders versus antidepressant non-responders). Multivariable logistic regression analysis was used to predict factors of perinatal depression (PND)/ postpartum depression (PPD), antidepressant use and antidepressant positive response in PND/PPD.

**Results:**

PND/PPD individuals had higher rates of mental health visits. No predicting factor for developing PND/PPD was shown. Antidepressant users were significantly older with insurance and recent health checkups/ mental visits. Being below the poverty level and having some health care accessibility are predictors for being on antidepressants. Recent non-illicit drug use is a predictor for PND/PPD symptom improvement while on antidepressants.

**Conclusion:**

The group of those with social-economic disadvantages was more likely to be on antidepressants for PND/PPD. Illicit drug users were less likely to show improvement with antidepressants. The safety and efficacy of antidepressant use during this period is controversial. More studies need to focus on the barriers involving antidepressant treatments, the safety and outcomes of antidepressants for PND/PPD management.

## INTRODUCTION

In the United States (US), depression places a major disease burden on those who are affected, their families and society. That burden has been especially reported during the perinatal and postpartum periods, with a prevalence ranging from 10% to 19.2% [1-5]. Studies of low-income and teenager mothers have reported a higher rate of depression symptoms at 40%-60% [6]. Due to help-seeking barriers, such as social and cultural stigma [7, 8], the real prevalence of perinatal depression (PND) and postpartum depression (PPD) might have been higher. Untreated depression during pregnancy leads to increased suicidal thoughts, miscarriage, low birth weight, preterm birth and negative effects on infants’ health (increased irritability, fewer facial expressions and being at risk for developmental delays) [9]. PPD might also have negative effects on infant development [6]. Thus, the proper management of PND and PPD is essential during this period.

Although most women during the pregnancy and postpartum periods report preferring non-pharmacotherapy treatment [10], arguably, the controversial use of antidepressants for PND and PPD increased threefold between 2002 and 2010 [11, 12]. The American College of Obstetricians and Gynecologists guidelines recommend antidepressants only for severe perinatal depression. Patients’ treatment responses and their degrees of comfort with initiating or discontinuing antidepressants also need to be considered [12]. In the existing literature, the risks of antidepressants on fetuses include miscarriage, birth defects, neonatal symptoms, irritability and cardiac malformations [13]. However, most antidepressants’ side effects were observed from observational studies and case studies with many confounding factors that could influence the studies’ results; thus, the safety and the potential, [15]. Believed to cause no fetal harm, non-pharmacotherapy treatment is preferred by many pregnant women, and physicians recommend it for mild to moderate depression [9, 12]. However, its costs, uncertain availability and questionable quality of delivery might prevent patients from achieving optimal outcomes with it [14, 15].

Even though a high need exists for improving PND and PDD management, as well as the understanding of these conditions, the use of antidepressants during the perinatal and postpartum periods and factors that influence antidepressant treatment success are still understudied. The prevalence studies in the literature showed an association between PPD-PNP and minority status as well as low socioeconomic status, low educational level and low social support. These studies’ limitations had small sample sizes, localized study sites and a special-population focus. Thus, the prevalence of the PND and PPD that they reported might not have been accurate or nationally representative [16-18]. Moreover, data regarding the factors associated with antidepressant users during the pregnancy and postpartum periods are lacking [19, 20]. Factors that affect PND and PPD improvement in antidepressant users are understudied. Limited data showed that minorities are less likely to have a robust response to antidepressants, through mitigation with a less-advantaged social status [21]. Given these disparities in the current knowledge regarding this topic, the objectives of this study are to utilize the National Health and Nutrition Examination Survey (NHANES) data to investigate the factors associated with PND and PPD nationally, to identify the characteristics of antidepressant users for PND and to analyze the associated factors that determine the improvement of depression in antidepressant users.

## MATERIAL AND METHODS

### Survey Design

Conducted by the National Center for Health Statistics, the NHANES is a cross-sectional and nationally representative survey sampling from the non-institutionalized U.S. population, used to assess the health and nutritional statuses of U.S. adults and children. The sample is selected to represent people of all ages in the U.S. population, with a realistic representation of all ethnicities. A combination of interviews and physical examinations, the survey is unique, offering the ability to report previously undiagnosed conditions and known conditions based on respondents’ responses [22]. Information on different disease risk factors, lifestyles, mental health, medication use and reproductive health has also been collected through the surveys. In this study, the data from the cohorts of 2007-2008, 2009-2010 and 2011-2012 were extracted and analyzed.

### Definitions of Inclusion and Exclusion Criteria and Variables

The sample for analysis included all NHANES adult pregnant women and women within the 18 months of the postpartum period. The depression status was defined as having a Patient Health Questionnaire (PHQ-9) (a validated tool for detecting depression signs and symptoms in this population) [9] score of at least 5 or being on antidepressant medications. The PHQ-9 scores were integrated in the NHANES survey. Antidepressant use was determined from the files. Several studies in the published literature utilized the PHQ-9 scores as a tool for depression diagnosis [23-25]. In addition, there was a high correlation between the depression diagnosis clinical diagnosis and PHQ-9 scores [26]. Those with the PHQ-9 scores met the criteria for depression had a high mortality rate in the long term [26, 27]. Therefore, the use of PHQ-9 score to determine the depression status was reasonable in this retrospective study given the availability of variables in NHANES data. The PND and PPD statuses were determined by matching the sequence respondent numbers (SEQNs) of the pregnant and 18-month-postpartum groups as well as the depression-status group.

Taken from different NHANES files, the variables of age, ethnicity, education levels, marital status and ratio of family income to poverty, the availability of health insurance and prescription coverage, recent health checkups, recent mental health visits and recent illicit drug use were used to analyze the differences and associations in this study. All of the files were matched by the unique individual SEQNs to determine the characteristics of the study groups. The three study groups that were analyzed and compared were (1) the perinatal/postpartum group with and without depression, (2) antidepressant users and non-antidepressant users with PND/PPD and (3) antidepressant users with and without normal PHQ-9 scores. A normal PHQ-9 score is less than 5.

### Statistical Analysis

The statistical package SAS 9.3 was used for all statistical data management and analyses. Binomial tests were utilized to compare the distribution differences among the groups, for each study aim. Chi-square tests, Fisher’s exact tests and ANOVA tests were used to elicit the differences in ethnicity, education level (at least high school graduate *vs* did not graduate), marital status (living with *vs* without spouses), availability of health insurance (yes *vs* no), prescription coverage (yes *vs* no), health care accessibility (recent visit *vs* no recent visit, and recent mental visit *vs* no recent mental visit), antidepressant use (yes *vs* no) and illicit drug use (yes *vs* no) among the three study groups. Differences in age, marital status and ratio of family income to poverty among the study groups were analyzed by the T-test and Mann-Whitney-Wilcoxon Test.

**Fig. (1):**
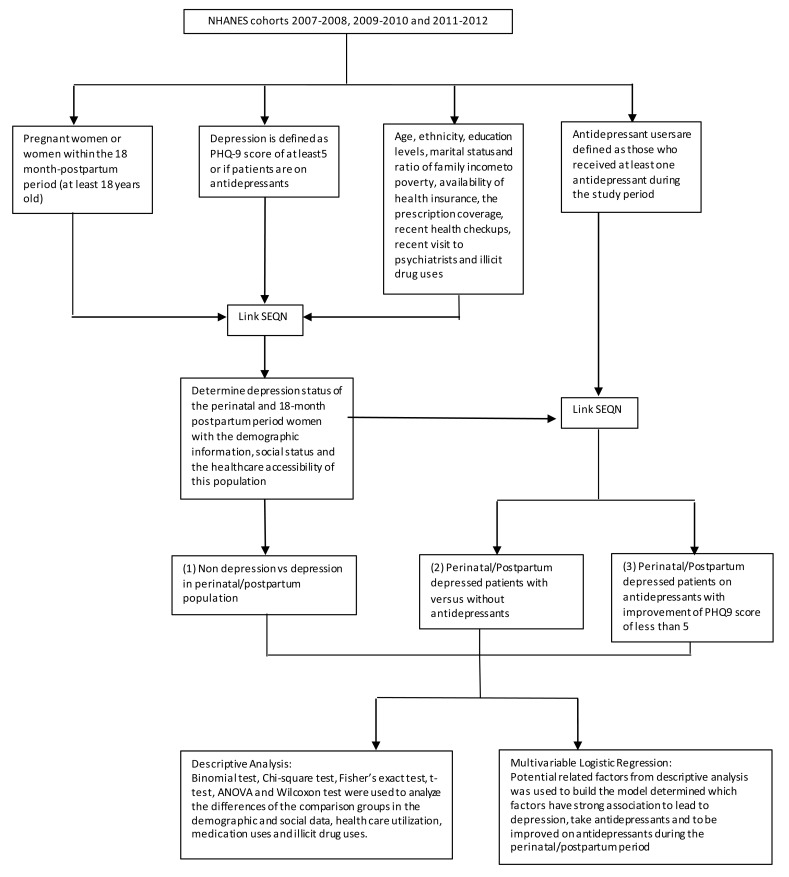
Study Methodology Flow Chart.

Multivariable logistic regression analyses were used to examine the predicting characteristics of PND and PPD, antidepressant users during the perinatal/postpartum periods and antidepressant users with normal PHQ-9 scores. The characteristics that were used for the model include age (<25, 25-29, 30-34, >34), ethnicity (non-Hispanic White, Hispanic, non-Hispanic Black, others), poverty rate (above or below the poverty rate), educational level (at least high school degree or no high school degree), marital status (lived with partner or no partner), insurance availability (yes or no), and recent health checkups (yes or no). The P-values of <0.05 were considered to be significant. The study design is illustrated in (Fig. **1**).

## RESULTS

When it came to determine the differences between the PND/PPD and no-depression groups in the analysis, a total of 492 respondents met the study criteria during the study period, with 216 participants (43.90%) meeting the depression criteria. No difference in ethnicity was found between the depression group and the non-depression group (p=0.4973). No statistically significant difference was found in average age, ratio of family income to poverty, educational level, availability of partners, availability of insurance, availability of prescription coverage, accessibility of health care services and illicit drug use between the group with and without depression. More recent mental health visits were discovered in the depressed group (17.13% vs 4.35%, p=0.001). The result of this part is summarized in Table (**1**). No characteristics were strongly associated with the increase in the risk of developing depression, based on the result of the multivariable logistic regression (Table **2**).

With regard to determining the differences between antidepressant users and non-antidepressants users in the analysis, the distribution of antidepressant users and the non-antidepressant users was similar (48.15% vs 51.85%; p=0.634). The list of used antidepressants was summarized in Table (**3**). No statistically significant difference was found in ethnicity, ratio of family income to poverty, educational level, availability of partners, availability of prescription coverage and illicit drug use between the two groups. Antidepressant users were significantly older and were more likely to have health insurance, recent health checkups and recent mental health visits than were those who were depressed and not on antidepressants (29.05±6.03 *vs* 27.14±5.26, p=0.0140; 90.38% *vs* 63.39, p<0.001; 98.08% *vs* 81.25%, p<0.0001; 29.81% *vs* 5.36%, p<0.0001, respectively). The results from this analysis are summarized in Table (**4**). The multivariable logistic regression is shown in Table (**5**). Most of the antidepressant users were below the poverty rate (p=0.0376), had health insurance (0.0005) and had just had recent health checkups (p=0.0148).

When it came to determine the differences between the antidepressant users achieving normal PHQ-9 scores (<5) and those not achieving normal PHQ-9 scores in the analysis, a total of 56 respondents had available data for antidepressant use and the PHQ-9 scores for the analysis. The proportion between the normal and abnormal PHQ-9 groups was similar (57.14% *vs* 42.86%, respectively; p=0.3497). No statistically significant differences were discovered among the two groups in ethnicity, age, ratio to family poverty, educational level, marital status, availability of insurance, prescription coverage and recent health visits. The respondents with normal PHQ-9 scores had fewer recent mental health visits than those with abnormal PHQ-9 scores (15.63% *vs* 50%, respectively; p=0.0056). Those with abnormal PHQ-9 scores had higher rates of recent illicit drug use than did the others (66.67% *vs* 31.82%, respectively; p=0.0281) (Table **6**). The multivariable logistic regression model of this aim showed that no illicit drug use was a predictor for achieving the normal PHQ-9 scores of antidepressant users during the perinatal and postpartum periods (p=0.0429) (Table **7**).

## DISCUSSION

Based on an analysis of the prevalence of depression and the factors associated with depression, the prevalence of depressed women during the pregnancy and postpartum periods in this study was higher than that in other studies (43.90%). Other studies excluded those who were treated and were stable on antidepressants or other forms of treatment. Different instruments used to detect depression symptoms can lead to different rates of depression in different studies [28]. Moreover, the populations in other studies were not representative of the entire U.S. population as the population in NHANES was, and some patients went underdiagnosed. Some studies included only the postpartum period for a short amount of time (3-12 months) [29, 30]. However, the literature stated that the rate of PPD and its negative impacts can still be significant during the 18-month postpartum period [31, 32]. Thus, with a longer postpartum time, as in this study, a higher rate of depression can be detected. In past published studies, patients did not tend to answer the survey honestly due to the presence of healthcare providers/ investigators and the fear of being diagnosed with depression, a mental illness with certain level of stigma, which might have recorded permanently in the medical history. In the NHANES survey setting, the participants might have responded more honestly to the PHQ-9 questions because there was no risk of being labeled as people with mental illnesses. Thus, the prevalence of the PND and PPD in other studies was lower than it was in this study.

The depression group had a higher rate of recent mental health visits, reflecting the reality that depression patients need follow up from psychiatrists. In the multivariable logistic regression, factors such as ethnicity, age, educational level, marital status, availability of insurance and recent health checkups were not associated with the increased risk of having depression. The results of this study are different from those of other studies, in which minorities and those with socioeconomic disadvantages had higher rates of depression than non-hispanic whites and those of higher socioeconomic statuses did [13, 16-18], as in many studies, the populations were not nationally representative. They were often localized or sub populations/special populations. Moreover, NHANES surveys can report undiagnosed conditions [22]; thus, a population that showed symptoms of depression through PHQ-9 scores but was not screened or detected by health care physicians emerged from the NHANES study population. This study showed no association of depression and disadvantaged socioeconomic status, with depression correlating with high-quality studies that demonstrated that low socioeconomic status, low level of education, lack of spousal support and lack of health care accessibility have a weak association for the increased risk of depression [33]. Based on this study, the risk of having depression during the perinatal and postpartum periods for U.S. women is equal for people of all ethnicities and socioeconomic statuses.

Regarding the analysis of taking antidepressants for PND, even though no differences between the proportion of antidepressant users and non-users were detected, the number of antidepressant users might have been higher due to missing information on the self-reported surveys. The prevalence of using antidepressants in this study was much higher than in previous studies, in which the prevalence of using antidepressants during the perinatal and postpartum periods ranged from 3.1% to 13.4% [19, 34-36]. In previous studies, the entire pregnant and postpartum populations were investigated, and the researchers did not focus on those with depression symptoms as our study did. Thus, our study is more prevalent specifically for the PND and PPD population. Most antidepressants were selective serotonin reuptake inhibitors (SSRI) and corresponded with current guideline recommendations to use SSRI because this drug class has the most information on safety for PND and PPD [12]. Antidepressant use in those with PND and PPD often posed an uncertain safety risk to the fetus and to infants despite the recommendations from different guidelines. With the high rate of using antidepressants, the outcomes of antidepressants on the mothers and infants will need to be investigated thoroughly.

No statistically significant difference was detected in ethnicity between antidepressant users and non-antidepressant users. In some studies, non-Hispanic Whites were more likely to be on antidepressants during the perinatal period [19, 37]. However, other studies concluded that ethnicity did not determine the difference to take or not to take antidepressants during the perinatal period [38, 39], and some results even showed that non-whites were more likely to be on antidepressants during this period [40]. Thus, there was even conflict in the literature when it came to drawing the conclusion of the results. In this study, due to the survey characteristics (the study was more nationally representative than other past studies were), the result might have reflected the reality of the nation. However, there was still a possibility of missing data since the medication data were partly self-reported. Thus, good prospective studies will need to be done to confirm the final results.

When it came to investigating the differences between antidepressant users and non-antidepressants users in the analysis, antidepressant users were significantly older (p = 0.044) than non-antidepressant users were. Older patients were more likely to try different alternative treatments that did not lead to the resolution of depression symptoms in the past. Thus, they had to be on antidepressants. Antidepressant users had higher rates of having health insurance, recent health checkups and recent mental health service visits than did non-antidepressant users. Thus, accessibility to health care increased the rate of antidepressant users in the study population.

The multivariable logistic analysis showed that the factors that strongly predict the use of antidepressants in the PND and PPD population included being below the poverty rate, having health insurance and having recent health checkups. Poverty was a factor for being on antidepressants because non-pharmacotherapy might not be covered by health insurance, and the cost is not affordable to many people. Having insurance and recent health checkups as predictors of antidepressant use demonstrated that having accessibility to health care is a strong predictor for being on antidepressants. It is unclear whether physicians overprescribed antidepressants to those with low socioeconomic status; future studies must focus on this topic.

When it came to comparing antidepressant users with normal PHQ-9 scores and those without normal PHQ-9 scores, no differences between the proportion of the normal– and abnormal–PHQ-9–score groups led to the questionable effectiveness of antidepressants for PND and PPD. The finding that ethnicity had no impact on the outcomes of antidepressant users in this study was consistent with findings in the literature [41]. Those with normal PHQ-9 scores had significantly fewer recent mental health visits and significantly less recent illicit drug use than did those who did not achieve normal scores. It is reasonable for stable antidepressant users to have less frequent checkups with their psychiatrists. Illicit drug use is one of the factors that contributes to the risk of depression. However, this risk is not consistent in the literature [42, 43]. In the multivariable logistic analysis, non-illicit drug users demonstrated as a factor that predicted depression symptoms’ improvement in antidepressant users. Illicit drug users often had uncertain degrees of medication compliance and complex mental histories; thus, they might have become resistant to treatment, such as that involving antidepressants. More studies must concentrate on the management of PND and PPD in illicit drug users.

The study includes some typical limitations of a retrospective survey study. There are missing data about prescription drug coverage, depression history and illicit drug use. A thorough analysis could not be completed on some of the variables. The utilization of the PHQ-9 survey to diagnose depression in the past published literatures was common, and the PHQ-9 survey built in the NHANES data sources was an excellent tool for the screening of depressive symptoms. However, the confirmed diagnoses were not included in the survey; therefore, the prevalence of depression might have been different. Respondents potentially did not answer honestly in the PHQ-9 and the antidepressant use survey. As a result, the prevalence of PND/PPD and antidepressant use might have been higher than detected in the study. The use of non-pharmacotherapy was not recoded; thus, it was unsure how non-pharmacotherapy might have had effects on PND/PPD, antidepressant use and improving the depression symptoms of antidepressant users. Since the medication data were partly self-reported, the investigation of the differences between antidepressant users and non-antidepressants users was challenging. However, this study’s methodology and results of the comparison between the antidepressant users and non-antidepressant users can be utilized as references for future studies with more complete responses. Thorough publicly available data sources with the emphasis on antidepressant utilization as well as perinatal and postpartum health will need to be developed in the future for national research purposes. The NHANES data is free and publicly available for researchers who are without access to large paid data. Therefore, outcomes from NHANES data like this study can serve as a preliminary investigation for the knowledge gaps in underserved population and highlight the lack of qualified open access data.

The study also featured many strengths. NHANES data are representative of the entire U.S. population compared to other studies in which the study populations were very specific. Moreover, those undiagnosed by health care professionals with PND and PPD were also included in the analysis of this study. Different from previous studies, this study focused specifically on antidepressant use in PND and PPD, while past studies including all pregnant women might have underestimated the prevalence of antidepressant use in the depressed population. This study highlighted the need for more research to understand the pattern of using antidepressants in the PND and PPD population. The study also analyzed the characteristics of those who were more likely to respond positively to antidepressants. The result highlighted that illicit drug users would be less likely to improve compared to others. Therefore, individualized PND/PPD treatment plans must be developed for illicit drug users due to potential treatment resistance.

## CONCLUSION

No differences in the ethnicities or social economic statuses between those with and without depression were detected. No factors can predict the risk of PND and PPD. However, about half of PND and PPD women were on antidepressants, with uncertain safety for fetuses/infants. Whether the physicians overprescribed perinatal/postpartum women will need to be investigated. Being below the poverty rate and having some accessibility to health care are predicting factors for antidepressant use in PND and PPD. Ethnicity was not a predictor for being on antidepressants or having a positive response to antidepressants. The treatment for PND and PPD in recent illicit drug users will have to be developed because of the likeliness of developing antidepressant resistance. Future prospective research regarding the most optimal care in PND and PPD must be done to generate the most optimal treatment plan for this vulnerable population.

## Figures and Tables

**Table 1:** Comparison of characteristics between depression versus no-depression group (N=492).

		**Depression versus No Depression**		
		Depression	No Depression	P-Value
		n=216 (43.90%)	n=276 (56.10%)	0.0068
Age (years) (Average ± Standard deviation)		28.06 ± 5.71	28.62 ± 6.00	0.2891
Ethnicity (Number (column %))	Non-Hispanic White	71 (32.87%)	89 (32.25%)	0.4973
	Hispanic	80 (37.04%)	111 (40.2)	
	Non-Hispanic Black	44 (20.37%)	59 (21.38%)	
	Others	21 (9.72%)	17 (6.16%)	
Ratio to family poverty (Average ± Standard deviation)		2.02 ± 1.57	1.93 ± 1.55	0.5203
Educational level (Number (%))	At least high school graduate or equivalent	166 (76.85%)	204 (73.91%)	0.4538
	No high school graduate	50 (23.15%)	72 (26.09%)	
Marital status (Number (%))	Lived with partner	164 (75.93%)	206 (74.64%)	0.7426
	Did not live with partner	52 (24.07%)	70 (25.36%)	
Insurance (Number (%))	Yes	165 (76.39%)	198 (71.74%)	0.2445
	No	51 (23.61%)	78 (28.26%)	
Prescription coverage (Number (%))^a^	Yes	155 (93.94%)	181 (90.95%)	0.2874
	No	10 (6.06%)	18 (9.05%)	
Recent health checkups (Number (%))	Yes	193 (89.35%)	230 (83.33%)	0.0564
	No	23 (10.65%)	46 (16.67%)	
Recent mental health visits (Number (%))	Yes	37 (17.13%)	12 (4.35%)	<0.0001
	No	179 (82.87%)	264 (95.65%)	
Illicit drug use (Number (%))^b^	Yes	74 (47.44%)	98 (42.98%)	0.3887
	No	82 (52.56%)	130 (57.02%)	

^a^ A total of 128 respondents were missing information for the analysis of the depression versus no-depression groups; ^b ^a total of 108 subjects were missing information for the analysis of the depression versus no-depression groups.

**Table 2:** Multivariable logistic regression with predictors of PND/PPD (N=492) of pregnant and 18-month postpartum women.

Predictor		**Depression**	
		**OR (95% CI)**	**P-Value**
Age	<25	1.39 (0.84, 2.28)	0.1657
	25-29	Ref	
	30-34	1.36 (0.80, 2.31)	
	>34	0.80 (0.45, 1.412)	
Ethnicity	Non-Hispanic White	Ref	0.4589
	Hispanic	0.93 (0.59, 1.45)	
	Non-Hispanic Black	0.92 (0.54, 1.56)	
	Others	0.93 (0.60, 1.45)	
Below the poverty rate	Yes	Ref	0.2206
	No	1.29 (0.86, 1.92)	
Educational level	At least high school graduate or equivalent	Ref	0.7588
	No high school graduate	0.93 (0.59, 1.45)	
Marital status	Lived with partner	Ref	0.907
	Did not live with partner	0.97 (0.62, 1.53)	
Insurance	Yes	Ref	0.5856
	No	0.88 (0.56, 1.38)	
Recent health checkups	Yes	Ref	0.1807
	No	0.67 (0.37,1.21)	
Illicit drug use	Yes	_	_
	No	_	_

**Table 3:** List of antidepressants used in 2007-2012 NHANES pregnant and 18-month postpartum women.

**Drug name**	**Frequency**	**Percent (%)**
**Amitriptyline**	5	4.81
**Bupropion**	14	13.46
**Citalopram**	14	13.46
**Doxepin**	1	0.96
**Duloxetine**	5	4.81
**Escitalopram**	11	10.58
**Fluoxetine**	10	9.62
**Imipramine**	2	1.92
**Mirtazapine**	2	1.92
**Nortriptyline**	1	0.96
**Paroxetine**	4	3.85
**Sertraline**	15	14.42
**Trazodone**	12	11.54
**Venlafaxine**	8	7.69

**Table 4:** Comparison of characteristics between antidepressant users versus non-antidepressant users (N=216).

		**Antidepressant Users versus Non-Antidepressant Users**		
		Antidepressant Users	Non-Antidepressant Users	P-Value
		n=104 (48.15%)	n=112 (51.85%)	0.634
Age (years) (Average ± Standard deviation)		29.05±6.03	27.14±5.26	0.014
Ethnicity (Number (column %))	Non-Hispanic White	29 (27.88%)	42 (37.50%)	0.1365
	Hispanic	44 (42.31%)	36 (32.14%)	
	Non-Hispanic Black	18 (17.31%)	26 (23.21%)	
	Others	13 (12.50%)	8 (7.14%)	
Ratio to family poverty (Average ± Standard deviation)		2.20±1.67	1.87±1.46	0.1303
Educational level (Number (%))	At least high school graduate or equivalent	83 (79.81%)	83 (74.11%)	0.321
	No high school graduate	21 (20.19%)	29 (25.89%)	
Marital status (Number (%))	Lived with partner	82 (78.85%)	82 (73.21%)	0.333
	Did not live with partner	22 (21.15%)	30 (26.79%)	
Insurance (Number (%))	Yes	94 (90.38%)	71 (63.39%)	<0.0001
	No	10 (9.62%)	41 (36.61%)	
Prescription coverage (Number (%))^a^	Yes	88 (93.62%)	67 (94.37%)	0.8417
	No	6 (6.38%)	4 (5.63%)	
Recent health checkups (Number (%))	Yes	102 (98.08%)	91 (81.25%)	<0.0001
	No	2 (1.92%)	21 (18.75%)	
Recent mental health visits (Number (%))	Yes	31 (29.81%)	6 (5.36%)	<0.0001
	No	73 (70.19%)	106 (94.64%)	
Illicit drug use (Number (%))^b^	Yes	33 (51.56%)	41 (44.57%)	0.3893
	No	31 (48.44%)	51 (55.43%)	

^a^ A total of 51 missing respondents in antidepressant-use versus non-antidepressant-use groups; ^b ^a total of 60 missing respondents in antidepressant-use versus non-antidepressant-use groups

**Table 5:** Multivariable logistic regression with predictors of taking antidepressants (N=216) of pregnant and 18-month postpartum women.

Predictor		**Antidepressant Use**	
		**OR (95% CI)**	**P-Value**
Age	<25	0.99 (0.42, 2.34)	0.1102
	25-29	Ref	
	30-34	2.2 (0.90, 5.36)	
	>34	2.28 (0.83, 6.31)	
Ethnicity	Non-Hispanic White	Ref	0.196
	Hispanic	1.88 (0.91, 3.90)	
	Non-Hispanic Black	1.40 (0.58, 3.40)	
	Others	3.02 (0.91, 10.02)	
Below the poverty rate	Yes	Ref	0.0376
	No	0..49 (0.25, 0.96)	
Educational level	At least high school graduate or equivalent	Ref	0.0725
	No high school graduate	0.50 (0.24, 1.065)	
Marital status	Lived with partner	Ref	0.4256
	Did not live with partner	0.73 (0.33, 1.59)	
Insurance	Yes	Ref	0.0005
	No	0.21 (0.09, 0.51)	
Recent health checkups	Yes	Ref	0.0148
	No	0.13 (0.03, 0.67)	
Illicit drug use	Yes	_	_
	No	_	_

**Table 6:** Comparison of characteristics between abnormal PHQ-9 scores versus normal PHQ-9 scores group (N=56).

		**Abnormal PHQ-9 versus Normal PHQ-9**		
		Abnormal PHQ-9	Normal PHQ-9	P-Value
		n=24 (42.86%)	n=32 (57.14%)	0.3497
Age (years) (Average ± Standard deviation)		28.67±6.01	29.13±5.23	0.762
Ethnicity (Number (column %))	Non-Hispanic White	7 (29.17%)	9 (28.13%)	0.711
	Hispanic	8 (33.33%)	15 (46.88%)	
	Non-Hispanic Black	4 (16.67%)	4 (12.50%)	
	Others	5 (20.83%)	4 (12.5%)	
Ratio to family poverty (Average ± Standard deviation)		2.23±1.79	2.47±1.77	0.7839
Educational level (Number (%))	At least high school graduate or equivalent	19 (79.17%)	23 (71.88%)	0.5329
	No high school graduate	5 (20.83%)	9 (28.13%)	
Marital status (Number (%))	Lived with partner	18 (75.00%)	25 (78.13%)	0.784
	Did not live with partner	6 (25.00%)	7 (21.88%)	
Insurance (Number (%))	Yes	21 (87.50%)	30 (93.75%)	0.417
	No	3 (12.50%)	2 (6.25%)	
Prescription coverage (Number (%))^a^	Yes	18 (85.71%)	28 (93.33%)	0.637
	No	3 (14.29%)	2 (6.67%)	
Recent health checkups (Number (%))	Yes	24 (100%)	30 (93.75%)	0.5013
	No	0 (0%)	2 (6.25%)	
Recent mental health visits (Number (%))	Yes	12 (50%)	5 (15.63%)	0.0056
	No	12 (50%)	27 (84.38%)	
Illicit drug use (Number (%))^b^	Yes	12 (66.67%)	7 (31.82%)	0.0281
	No	6 (33.33%)	15 (68.18%)	

Total of 16 missing respondents in group with abnormal PHQ-9 scores versus normal PHQ-9 scores

**Table 7:** Multivariable logistic regression with predictors of improving while on antidepressants (N=40) of pregnant and 18-month postpartum women.

Predictor		**Positive Antidepressant Response**	
		**OR (95% CI)**	**P-Value**
Age	<25	1.27 (0.09, 17.56)	0.4
	25-29	Ref	
	30-34	6.376 (0.576, 70.54)	
	>34	1.89 (0.22, 16.43)	
Ethnicity	Non-Hispanic White	Ref	0.93
	Hispanic	0.51 (0.06, 4.72)	
	Non-Hispanic Black	1.03 (0.06, 16.77)	
	Others	0.67 (0.04, 10.32)	
Below the poverty rate	Yes	Ref	0.73
	No	1.10 (0.15, 8.19)	
Educational level	At least high school graduate or equivalent	Ref	0.49
	No high school graduate	0.34 (0.03, 4.39)	
Marital status	Lived with partner	Ref	0.73
	Did not live with partner	0.65 (0.06, 7.38)	
Insurance	Yes	Ref	0.62
	No	0.63 (0.05, 8.38)	
Recent health checkups	Yes	_	_
	No	_	_
Illicit drug use	Yes	Ref	0.0429
	No	0.16 (0.03, 0.94)	

## LIST OF ABBREVIATIONS

NHANES: = National Health and Nutrition Examination Surveys
PHQ-9: = Patient Health Questionnaire
PND: = Perinatal depression
PPD: = Postpartum depression
SAS: = Statistical Analysis Software
SEQN: = Sequence respondent number
